# Synergistic Growth in Number of Diagnostic and Interventional Radiology Matches at Pennsylvania State College of Medicine After 2016

**DOI:** 10.7759/cureus.8949

**Published:** 2020-07-01

**Authors:** Surav M Sakya, Trishae Winters, Sydnie Thomas, Mary L Dinh, David R Hallan

**Affiliations:** 1 Medicine, Penn State College of Medicine, Hershey, USA; 2 Neurosurgery, Penn State Milton S. Hershey Medical Center, Hershey, USA

**Keywords:** medical residency, residency preparation, residency, residency application

## Abstract

In 2016, when interventional radiology (IR) separated from diagnostic radiology (DR), the future implications were unknown. The goal of this study is to investigate trends in DR and IR matches at Penn State College of Medicine (PSCOM) from 2011-2020, particularly before and after 2016. This retrospective study generated descriptive statistics and paired t-tests, finding a statistically significant difference in mean number of DR and IR matches before and after 2016. More specifically, the number of DR matches increased from 3.8 to 6.4 (p = 0.0004) and IR matches increased from 0 to 2 (p = 0.03). These trends suggest a synergistic growth in both specialties.

## Introduction

In recent years, interventional radiology (IR) transitioned to a separate residency program apart from diagnostic radiology (DR). The first integrated IR residencies were incorporated into the match process in 2016 [1]. With this transition, trends in radiology match data have changed. Both specialties are becoming increasingly popular and competitive [2]. Starting in 2020, IR residency training can occur through IR and DR programs [3]. Because of the novelty of IR and the multiple pathways available, it makes sense that popularity would spike in both DR and IR [2]. Due to its popularity, IR has also become an extremely competitive field to match into. According to results from the 2018 match, the integrated IR residency had 0.66 positions per US applicant, the lowest of any specialty, while DR had 1.43 positions per US applicant [4]. Given these recent studies in the literature, we wanted to investigate the match results for DR and IR at Penn State College of Medicine (PSCOM). The purpose of this study is to investigate the trends in DR and IR matches at PSCOM from 2011-2020 to see if the results reflect the current literature. We hypothesize that at PSCOM, both DR and IR will show an upward trend after 2016 due to the start of the integrated IR residency, and that DR will trend higher than IR due to the competitiveness of IR.

## Materials and methods

This study is a retrospective study of match data for DR and IR at PSCOM from the years 2011-2020, gathered from a handbook created by PSCOM medical students [5]. Publicly available data from the National Resident Matching Program were also compared with the PSCOM data [6,7]. We then generated descriptive statistics, including mean, median, and standard deviation, through statistical software R to help analyze the trends in both specialties at PSCOM before and after 2016, the year when the integrated IR residency was made available for one IR residency position each year at Penn State Health Milton S. Hershey Medical Center. Mean values were presented as ± significant difference with a 95% confidence interval. We performed a paired t-test to determine any significant differences between means (p < 0.05). Institutional Review Board (IRB) exemption was granted for this study.

## Results

The total number of PSCOM students who matched into DR from 2011-2020 was 51, and the total number of matches for IR was 10 (Table 1).

**Table 1:** Number of Diagnostic Radiology (DR) and Interventional Radiology (IR) Matches at Penn State College of Medicine by Year 2011-2020 The table shows the total number of matches into DR and IR, along with the number of matches by year. The number of matches trends upward for both DR and IR after 2016, with DR trending downward in 2020 and IR trending downward in 2019-2020.

Specialty	2011	2012	2013	2014	2015	2016	2017	2018	2019	2020	Total
DR	6	4	3	4	2	8	7	6	7	4	51
IR	0	0	0	0	0	1	4	3	1	1	10

The mean number of IR matches unsurprisingly was 0 ± 0 in 2011-2015 and jumped to 2 ± 1.41 in 2016-2020 after the start of IR matches in 2016 (Table 2). 

**Table 2:** Statistical Analysis of Diagnostic Radiology (DR) and Interventional Radiology (IR) Matches at Penn State College of Medicine (PSCOM) by Timeframe The table shows the descriptive statistics generated for the number of matches in DR and IR at PSCOM, including mean, median, and standard deviation. Statistics were calculated for the entire period of the study (2011-2020); statistics were also calculated for 2011-2015 and 2016-2020, since the integrated IR residency began in 2016. After 2016, the mean number of matches increased for both DR and IR.

Residency Program	Total (N)	Median	Mean ± SD (CI)	Difference Before vs. After Separate IR Residency Established in 2016
DR 2011-2020	51	5	5.1 ± 1.97 (3.69 to 6.51)	-
IR 2011-2020	10	0.5	1 ± 1.41 (-0.01 to 2.01)	
DR 2011-2015	19	4	3.8 ± 1.48 (1.96 to 5.64)	p = 0.0004, 95% CI (1.92 to 3.28)
DR 2016-2020	32	7	6.4 ± 1.52 (4.52 to 8.28)	
IR 2011-2015	0	0	0 ± 0	p = 0.03, 95% CI (0.24 to 3.76)
IR 2016-2020	10	1	2 ± 1.41 (0.24 to 3.76)	

**Figure 1:**
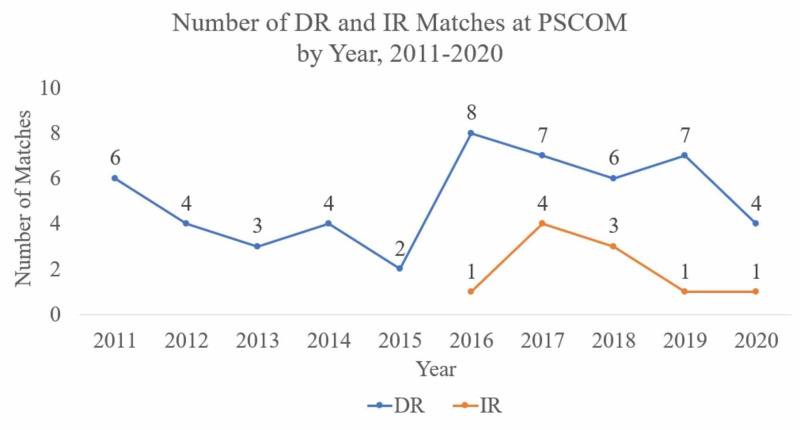
Graph of Diagnostic Radiology (DR) and Interventional Radiology (IR) Matches at Penn State College of Medicine (PSCOM) by Year 2011-2020 This graph compares the number of matches in both DR and IR at PSCOM, with DR in blue and IR in orange. Both specialties trend upward after 2016, with DR remaining stable until trending downward in 2020 and IR trending downward after 2017.

The number of matches for DR appear to trend upward in 2016 when IR matches began, with DR remaining mostly stable through 2019 before trending downward in 2020 and IR trending downward after 2017 (Figure 1).

## Discussion

The results appear to support our hypothesis. The mean number of matches significantly increased in both DR (3.8 to 6.4, p < 0.05) and IR (0 to 2, p < 0.05) after the start of integrated IR residencies in 2016, indicating an upward trend. The number of DR matches also trended higher than IR matches every year, although both specialties trended downward in 2020. It is unclear if these downward trends will continue, or if it is due to a small sample size of time. These downward trends may also reflect a stabilization of the demand for IR now that it is not as much of a novelty. Overall, the upward trend in both specialties is consistent with Yi et al., who found that both specialties are indeed becoming more popular, as well as the trends found in the national match data [2,6,7]. Although the number of DR matches has overall been decreasing nationally since 2011, matches spiked in 2016 and stabilized until 2019-2020; this might indicate that interest in DR also increased nationally after 2016 [6,7]. Furthermore, Goldman et al. found that the competitive nature of the application process for IR vs. DR had a negative impact on current radiology residents when choosing their radiology career, making it possible that competitiveness would dissuade students from choosing IR [8]. According to Umer et al., students interested in IR may opt for DR to increase their chances of matching, and then reconsider IR in the future [9]. This could be an explanation for the PSCOM match results, which are also reflected in the national data; the number of DR matches consistently trended higher than IR from 2016-2020, perhaps because DR is not as competitive, and it is possible to pursue IR through DR. An alternative explanation could be that there are more IR applicants than IR positions available, while DR has a higher number of positions available per applicant [4]. 

Limitations

The sample size of data is small; since the IR residency started in 2016, we were only able to compare DR and IR from the time period of 2016-2020. Students can opt out of the public match list at PSCOM, so the values reported may not be complete.

## Conclusions

When the integrated IR residency became a part of the match process in 2016, it generated new trends in both DR and IR matches. The purpose of this study was to investigate the trends in DR and IR matches at PSCOM from 2011-2020. At PSCOM, the number of DR and IR matches trended upward after 2016, and DR consistently trended higher than IR in every year. Overall, the results suggest a synergistic growth in the two specialties.
